# “It’s not a life of war and conflict”: experienced therapists’ views on negotiating a therapeutic alliance in involuntary treatment

**DOI:** 10.1186/s12991-019-0234-6

**Published:** 2019-06-21

**Authors:** Marius Prytz, Karina Natalie Harkestad, Marius Veseth, Jone Bjornestad

**Affiliations:** 1 Department of Clinical Psychology, University of Bergen, Christies Gate 12, 5012 Bergen, Norway; 2 Department of Social Studies, University of Stavanger, Stavanger, Norway

**Keywords:** Working alliance, Coercive treatment, Recovery, Qualitative research, Therapist perspective

## Abstract

**Background:**

Working alliances are considered to be essential to treatment, and they represent a robust predictor of positive treatment outcomes. In a working alliance, a patient and therapist agree upon treatment decisions, which can raise a series of challenges when patients are in involuntary treatment. The aim of this study was to research how therapists experience negotiating a working alliance with patients with serious mental illnesses who are subjected to coercive treatment.

**Methods:**

Using a qualitative approach, we conducted 10 semi-structured interviews with experienced therapists in a Norwegian mental health care setting. Transcripts were analysed using a team-based thematic analysis method.

**Results:**

Two interrelated major themes and five sub-themes were identified: (1) between coercion and care; (a) the ease of coercion, (b) the paradox of autonomy, and (c) the coercion as care; and (2) imperative treatment and interpersonal dilemmas; (a) this is happening between us and (b) when we do not meet in the middle.

**Conclusion:**

We conclude that the therapists exhibited a will to consider their patients’ goals and methods, but only when they were in agreement, and they ultimately made treatment decisions themselves. Further, patient autonomy seems to come second in therapist assessments of needs for care; consequently, we question to what degree the working alliance as a defined concept of mutual agreement is present in the involuntary treatment we investigated.

## Background

In psychological treatment, the establishment of a working alliance between the patient and therapist is essential [1]. Currently, a working alliance is understood as a bond between a patient and therapist involving elements of trust and acceptance [2], active cooperation and mutual agreement on treatment goals and methods [3–5]. Working alliances are widely considered a robust predictor of positive outcomes across psychotherapeutic treatment approaches [6, 7]. As a fundamental principle, a working alliance should be established with the patient’s consent as a collaborative enterprise [8]. In the treatment of people with severe mental illnesses, this principle is sometimes deviated from, implying involuntary treatment against the patient’s will [9, 10]. In Norway, involuntary mental health care can only be applied when the patient is a danger to him or herself or others, and consequently, when it appears to be the expedient option [8, 11]. In 2014, the Norwegian Patient Register (NPR) reported nearly 8000 coercive admissions to institutions [12] for a total of 500–600 individuals.

The current study is a contribution to and placed within the theoretical framework of research on therapeutic alliances. The literature on how therapists establish working alliances with patients in coercive treatment settings is generally scarce. However, working alliances with individuals with serious mental disorders, which represents the group most often admitted involuntarily, have been studied to a greater extent. Studies find it possible to develop and maintain a good therapeutic relationship with patients with psychosis and indicate that this is associated with recovery [13, 14]. In addition, therapists who consistently manage to establish a strong alliance with their patients enjoy better results than therapists who are not able to establish a strong alliance [15]. As such, it is of importance to examine how therapists experience establishing working alliances with those subjected to coercive treatment.

Individuals with first-hand experience with mental illness express a wish to be part of a process-oriented therapy approach towards recovery, which materialises in a focus that extends beyond symptom reduction [16, 17] and rather involves therapists expressing hope and a belief in the patient’s recovery [18]. Patients are seen as autonomous agents and collaborative partners, and they are expected to be active in the design of their own treatment [19, 20]. These fundamental principles are at the core of what we may call a recovery perspective [21, 22]. The person in treatment is here at the centre stage as the aim of care is shifted towards building a meaningful life in the community. These basic principles of what has been called recovery-oriented practice or person-centred care [23] also apply in cases of involuntary treatment [24]. However, this balance appears more challenging in involuntary treatment, as this form of treatment is often experienced as particularly demanding and in violation of patient autonomy [11, 25]. In addition, patients who are in involuntary treatment have certain expectations towards the treatment [7], and might perceive coercive admission as a terrifying experience [24]. Kaltiala-Heino et al. [26] found that coercive treatment often leads to negative emotions in the patient, in addition to negative expectations regarding treatment outcome. Use of involuntary mental health care complicates the establishment of a trustworthy relationship between the patient and therapist and may also cause the patient to want to use voluntary health services in the future even less [26, 27].

In the last decades, research on therapeutic alliances has focused on processes involved in alliance ruptures and their repairs [28, 29]. It is expected that a rupture in the working alliance occur [29]. This could be a small tension or a severe breakdown in the cooperative relationship between patient and therapist [28]. Coercive admission may make the establishment of a good working alliance challenging, because the starting point of the treatment might be perceived as an alliance rupture between patient and therapist [30]. The focus on repairing alliance ruptures may be seen as one of the most important assignments in psychotherapeutic courses, because ruptures challenge active cooperation and thus also recovery [28].

### Research question

How do therapists experience negotiating working alliances with patients with serious mental illnesses subjected to coercive treatment?

## Methods

### Design

In this qualitative study, we used a thematic analytic approach [31, 32] within an interpretative phenomenological framework [33]. In this study, the interpretative element implies that data were generated through a reflexive dialogue between participants and researchers. The phenomenological element suggests that significant knowledge was collected from individuals with first-hand knowledge of negotiating a working alliance with patients with serious mental illnesses subjected to coercive treatment. The central aim was to discover and interpret the meaning of such experiences within their broader contexts [34].

### Reflexivity

The interpretative phenomenological approach taken by the authors implies that we aim to explore and describe the views on establishing working alliances in involuntary treatment settings, but by doing so, we need to acknowledge that we are informed by our own experiential horizons [33]. All authors are clinical psychologists and practice as psychotherapists. The first and second authors were students at time of data collection, whilst the third and fourth authors are associate professors. We all share interest for working alliances, how involuntary treatment is conducted, and recovery-oriented practice.

### Sampling and recruitment

Participants were included when they met the criteria of being experienced clinical psychologists or psychiatrists working in hospitals in western Norway. Participants were identified using a snowball sampling technique [35]. First, we identified a few therapists by contacting relevant hospitals; thereafter, we asked participants again if they knew of other professionals working with people in involuntary treatment settings. Sixteen eligible candidates were contacted; of these, six individuals refused to participate. The sample size was decided based on the stability of findings [36], reviewed after 6 and 8 participants. We stopped recruiting after 10 participants because we considered the last 2 interviews not to contribute substantially new information. A total of 10 individuals were recruited, including 8 women and 2 men aged 40–66 years (*M* = 51, SD = 8.81). Four were clinical psychologists, and 6 were psychiatrists. The years of experience in their respective fields ranged from 8 to 38 years (*M* = 21.6, SD = 10.28). This included experience working with involuntary treatment in psychiatric hospitals for 1.5–12 years (*M* = 7.25, SD = 3.68). Specifically, their work experience included work in acute psychiatric departments, in psychiatric inpatient wards with short- and long-term hospitalisation and in outpatient clinics. In addition to being specialists of psychiatry or clinical psychology, they had received special education in cognitive behavioural therapy (*n* = 5), psychodynamic therapy (*n* = 2), psychosocial rehabilitation with psychosis (*n* = 2) and mentalisation-based therapy (*n* = 1). Three reported multiple special educations (i.e. both cognitive behavioural therapy and psychodynamic therapy) and four participants did not specify special education beyond their clinical specialisation.

### Data collection

Participants were interviewed between October 2017 and February 2018. A semi-structured interview guide (Appendix) [37] was developed by the authors based on the theoretical concepts of working alliance [3, 28] rooted in tasks, goals and bonds and in participants’ general experiences of treating patients in involuntary treatment settings (i.e. “Can you describe what it is like providing involuntary treatment to people with serious mental disorders?”, “Have you experienced any challenges in relation to agreeing on mutual goals?” and “Have you experienced alliance ruptures?”. The first and second authors (MP and KNH) performed 5 interviews each (of 45–75 min) conducted at either the participant’s workplace or at the University of Bergen. Informed consent was obtained. At the end of each interview, participants were invited to give further information not covered during the interview and they filled out a brief demographic form. The interviews were audio recorded and transcribed verbatim for the purpose of analysis.

### Data analysis

Data were analysed with a team-based approach to thematic analysis following six steps [31]:

1. We familiarised ourselves with the data by repeatedly reading the interview transcripts. Main impressions were recorded and discussed as the process moved forward.
2. Initial codes were generated and defined as the most basic segments of the raw data that represented meaningful aspects related to the research question. In collaboration, we discussed coding practices to reach agreement.
3. We searched for preliminary themes and sub-themes. All codes were analysed with the goal of identifying broader patterns of meaning. In this phase, the third author was included in further discussions.
4. Tentative themes were reviewed and discussed when they fit the codes and overall dataset. This occurred through a back and forward process through which we changed themes and sub-themes and found new themes.
5. After further discussion, themes were defined and named. The fourth author was asked to critically audit the themes. Finally, all authors agreed upon the overall analysis of the dataset.
6. We partnered in writing the article, ensuring that the findings were presented in a detailed and meaningful way in relation to the research question.

## Results

The analysis yielded two interrelated major themes and five sub-themes illustrating how the therapists experienced working alliances with patients subjected to involuntary treatment. The first major theme concentrates on agreeing upon treatment plans with patients whilst the second major theme focuses on relational aspects of treatment collaboration.

### Between coercion and care

The first major theme concentrates on how the therapists achieved agreement on the goals and content of treatment with their patients. This served as a meeting point between their clinical opinions and their patients’ wishes.

#### The ease of coercion—“Do you agree with me, or are you simply accepting my right to make decisions?”

All the therapists considered it feasible to achieve a working alliance with patients subjected to coercive treatment. The therapists usually viewed the contact aspect of each alliance as unproblematic, as patients often agreed upon the goals and content of the treatment.

The general experience is that this is unproblematic. Challenges that might arise because we administer involuntary treatment—which is a major intervention into another person’s life and freedom—are challenges that are managed and are not constant, and they do not present considerable challenges to contact. (Therapist 10)

Most therapists described themselves as authorities that executed power. They specified that they wished for and aimed at considering their patients’ views on treatment decisions. However, the final decision on what was included in each treatment plan was always theirs to make. Several therapists wondered if the asymmetric relationship between the therapist and patient constituted the very reason why the patients easily agreed to treatment plans whilst being subject to coercion:

When I consider the fact that we often are that authority figure they need to relate to, I think that it is quite astonishing how often it is possible to reach them and not get set aside, if I can put it like that. However, we often experience that too. Before I started [working] here, I mostly thought that this must be a life of war and conflict, but it is not truly. How strange it must seem. They sort of accept our right to exert that authority very often, so there doesn’t have to be much heat. Sometimes there is, but most often not. There is truly not much resistance. (Therapist 8)

Some participants, however, questioned whether their patients did not accept treatment plans but refrained from opposing their therapist:

I think the range of agreement is quite broad. Do you agree with me, or are you simply accepting my right to make decisions? Those are two different things, and many take this latter approach and don’t approve of coercion but one does not make a fuss and accept that decisions are being made on their behalf. (Therapist 8)

#### The paradox of autonomy—“You may choose to be seriously mental ill as long as I consider that you are consent”

All participants emphasised the patients’ autonomy in treatment. They always strived for as much patient autonomy as possible. At the same time, the participants were explicit that they continuously needed to appraise how much and which type of autonomy the patients could handle and based on which life domains, forms of functioning, levels of treatment consent and so on. These considerations usually ended with limitations in patient autonomy. We also found that therapists strove to have the patients actively participate in conversations about treatment, including those focused on treatment aspirations or goals for the future. This approach was viewed as an attempt to have patients own their ongoing treatment and care.

I am clear on the question “What is it that you want?” and believe that they should be allowed to decide. “What do you wish for? On what issues can we agree, what do you want help on?” (…) “How can we reach these goals together although we have a starting point from which we completely disagree?” (Therapist 10)

Many participants noted the importance of verbalising empathy when patients felt they had lost autonomy. Moreover, the participants conveyed that not all autonomy was lost in the treatment setting. This was also illustrated when some participants spoke of forms of autonomy the patients could exhibit in involuntary treatment settings, for example, on types of medication used or on the ability to control their own circadian rhythm.

When the patient fills criteria for involuntary treatment but based on the patient having a degree of decision-making freedom to say “Yes, but I choose to live my life even if I become psychotic or even if I have these voices, so it will be fine”, then this will be emphasized, and one should not administer involuntary treatment. You shall be allowed to choose to get worse, and you shall be allowed to choose to be seriously mentally ill as long as we consider them to have the competence to give informed consent. (Therapist 10)

#### Coercion as care—“He doesn’t understand; he cannot make that decision by himself”

The accommodation of patients’ wishes was viewed as particularly challenging in cases where patients exhibited a clear lack of insight and did not agree on treatment goals or methods. In these cases, it was made clear that the solutions to patients’ challenges were to be found within a context of coercive treatment. The process leading to this type of decision was characterised as a process that involved caring for the patient or protecting society.

You can draw parallels to patients with dementia or… I mean, there is something about… I mean, I think there is a sense of dignity. The system we now have, with involuntary treatment, I think it’s a very good system. See what it is like in other countries where you do not have it, where only criteria on safety are applied [for oneself or others], for example. I mean, they live on the street, they make fools of themselves. I have been abroad and seen schizophrenics walking around in dirty underwear shouting in the street day after day. Obviously we do not see this in Norway, because we believe that this is a person who is seriously ill, and this is where we have a capacity to give informed consent. He does not understand; he is unable to make that decision for himself. (Therapist 3)

All the therapists expressed a positive view on the use of coercive treatment. However, several of the informants pointed out that coercive treatment has negative consequences for people’s self-determination. The therapists also regularly questioned to what degree patients’ human rights were to be granted and the potentially offensive nature of such coercion.

Well, yes, I’m thinking that there is a great degree of political opposition to involuntary treatment, one talks about human rights and violations of personal boundaries and that is in a way completely fair. At the same time, there is something about this approach serving as a tool for society to provide necessary health services to people who will not ask for them and who are in a given position because of their illness. If one doesn’t view that as ethically sound, then it is difficult to be in this position, but I’m thinking that we’re making use of this approach even though society doesn’t always understand it, sort of… I also do not think it is as bad as some say it is… even though there are things that are hard to explain and justify. However, in individual cases this does seem reasonable, sensible and dignified, and it doesn’t have to be any more difficult than that. (Therapist 8)

Some therapists expressed that challenges faced, when applying involuntary treatment, could create a feeling of discomfort or unease. This could be because of a patient’s violent actions, yelling or heated discussions. These situations would be solved by making the patient feel safe and express empathy. Further, the therapist could point out the unacceptability in the actions. However, the therapists pointed out also these situations were manageable.

### Imperative treatment and interpersonal dilemmas

The second major theme illustrates how the therapists, when experiencing difficulty in reaching agreement on treatment goals, narrowed treatment down to the therapist–patient interaction. Sub-themes are divided into relational factors and treatment content.

#### This is happening between us—“We have to ensure that they understand that we want to help”

Most participants described the establishment of trust and safety as particularly important building blocks in involuntary treatment. They specified that to make progress in the course of treatment and to have a patient agree upon treatment goals, they need to make each patient feel safe and respected. Sometimes, the therapeutic relationship serves as the treatment itself due to a patient’s reluctance to converse about treatment goals or agree on assignments.

Different relational factors such as those related to explicit openness, honesty and respect were viewed as therapeutic tools used to help make patients feel safe in working towards a joint goal and towards recovery. The following quote summarises what several participants stated: “… show that I genuinely care.” (Therapist 10). Several pointed out that these factors were similar in voluntary treatment settings, but that they were especially important in involuntary treatment settings due to violations perceived by some patients.

Many participants described specific obstacles to establishing a good therapeutic relationship, including a patient’s paranoia towards the therapist. One approach to overcoming such obstacles involved discussing everyday topics such as music preferences or family relationships and noting something positive in the patient’s life. This common courtesy was viewed as beneficial in helping people feel safe and comfortable and hence in strengthening the therapeutic relationship.

… When they are very, like, vulnerable, we assure them that we are only looking to help. We point out something that is positive. When we see a person who is well groomed, who looks well, and who appears as if they are concerned with diet and exercise, we confirm those types of things. That means a lot for the alliance. Yes. How I experience the greeting… “You look well today”. *Laughs* These are ordinary things we all appreciate. (Therapist 4)

#### When we do not meet in the middle—“Sometimes we cannot make it work”

Although the majority of the participants mostly viewed the establishment of good working alliances as relatively unproblematic, many had experienced instances in which establishment proved very challenging. In such cases, it was often decided to remove the patient from involuntary treatment and to possibly re-admit the patient. In other cases, it was considered beneficial to approach the situation gently without pushing the patient. This approach was particularly preferred in cases of active psychosis. Here, many emphasised that it was most efficient to listen to psychotic delusions whilst at the same time spend time assisting with practical issues such as sleeping routines and leisure activities.

Sometimes we cannot make it work. One simply has to try… in the most gentle and sensitive way, to convey that “It is important that you cooperate now and that this may… your illness may get worse and it may end in readmission”. However, this can also be experienced as very threatening to them. Therefore, … one must repeat this statement and hope some of them embrace it. Some do, and some absolutely don’t. (Therapist 7)

## Discussion

In this study, we have presented an analysis of how 10 therapists experienced establishing a working alliance with patients in involuntary treatment settings, which resulted in the identification of two interrelated major themes and five sub-themes. How can we understand these findings and what implications do they hold for treatment and care?

### Modelling the relationship between coercion and care

**Fig. 1:**
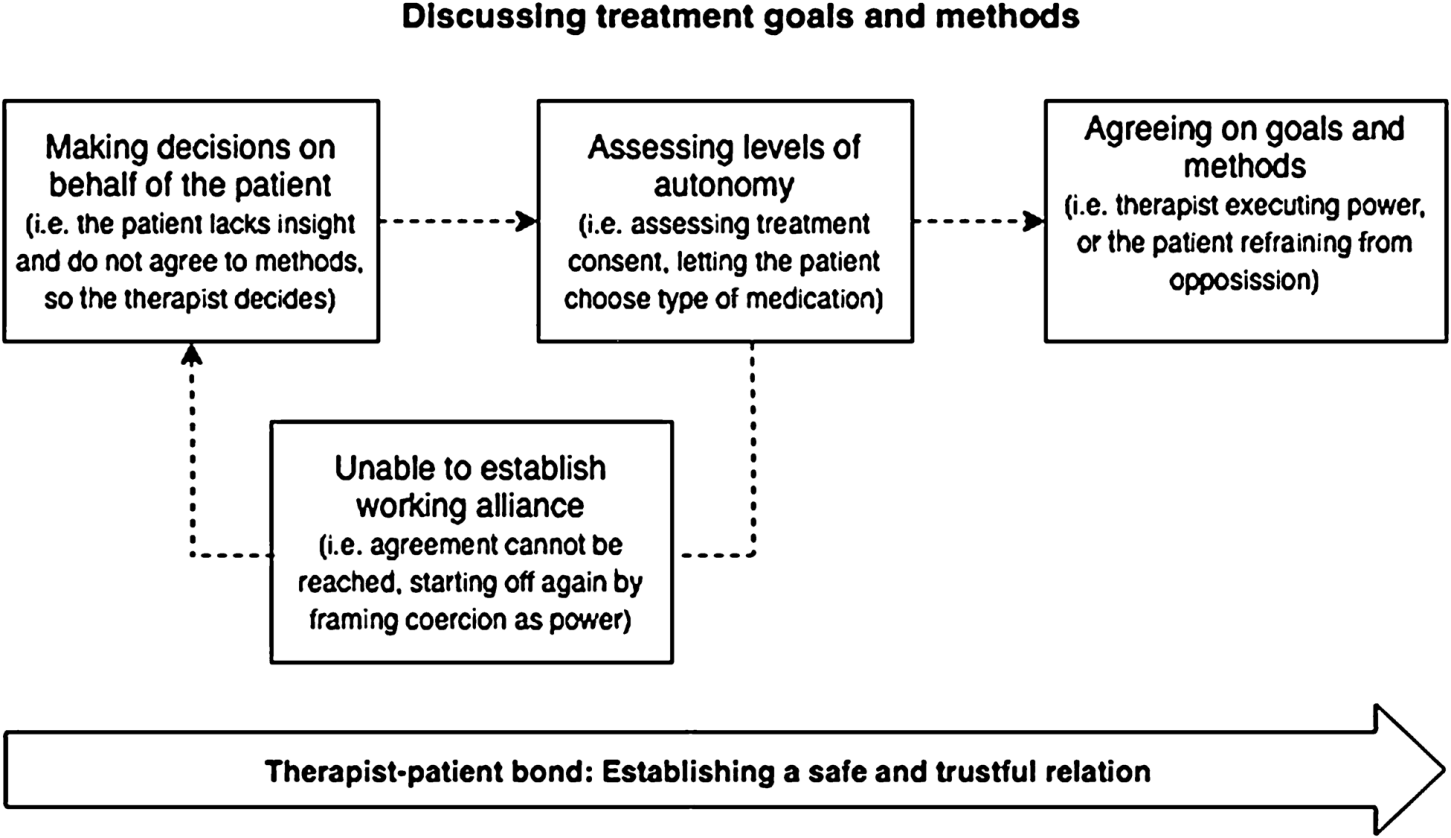
The figure illustrates the modelled relationship between coercion and care. The “linear” boxes, or “decision stages”, represent different factors or phases experienced on the way to agreeing or disagreeing over the course of treatment. “Therapist–patient bond” refers to the therapist–patient relationship and overlaps the bond component of working alliances. This process seems more independent of treatment discussions and is viewed more as a parallel process during treatment

We discovered a continuum between autonomy and coercion and observed how therapists alternated between these poles. Four (out of five) of these themes (“The ease of coercion”, “The paradox of autonomy”, “Coercion as care” and “When we do not meet in the middle”) arguably seemed to reflect different aspects of a linear interdependent process when discussing treatment goals and content. These “linear” themes are conceptualised into four possible “decision stages” in the model and represent different factors or phases experienced on the way to agreeing or disagreeing over the course of treatment. The stippled lines illustrate the treatment discussion might move from one box to the next, but could also be concluded in one of the earlier stages. In the box “Assessing levels of autonomy”, the result might be that treatment discussion does not succeed. Thus, treatment discussion might lead to what is described in the theme “When we do not meet in the middle”, which is conceptualised as “Unable to establish working alliance” and could be viewed as the stage where the therapist and patient do not agree. At the same time, this theme can also be seen as the start of a new process, where the therapist, as a result of recognising that agreement cannot be reached, changes his or her approach starting off again by framing coercion as power. The conversation continuously balances framing involuntary treatment as care or coercion. The last theme of this model, “This is happening between us”, conceptualised as “Therapist–patient bond”, refers to the therapist–patient relationship and overlaps the bond component of working alliances. This process seems more independent of treatment discussions and is viewed more as a parallel process during treatment. During treatment, the therapist focuses on helping the patient feel safe in the therapeutic relationship. When treatment is characterised by disagreement and conflict, the therapist instead focuses on relational factors without touching on the issue of treatment content. In doing so, the therapist brings treatment forward and may forge an agreement at a later stage. See Fig. 1 for our model.

### The unsolvable tension between autonomy and coercion and potential clinical implications

As one main finding, we discovered that the therapists characterised their experience of negotiating working alliances and reaching agreement as somewhat easy or manageable. The therapists did point out difficulties during treatment courses. However, the overall impression was that these disagreements were manageable. This was surprising, as research implies this might be a greater challenge [7, 11] and that the conceptual definition requires patient agreement, which is absent prior to and sometimes throughout involuntary treatment. The therapists exhibited a will to consider their patients’ goals and methods, but only when they were in agreement, and they ultimately made decisions themselves. This might be viewed as a tokenistic or artificial form of involvement as opposed to equal participation and thus may oppose basic elements of a working alliance. In interpreting our results in this way, we question to what degree the working alliance as a defined concept of mutual agreement is present in the involuntary treatment we investigated. However, working alliances still seem achievable, though in a different form than in voluntary treatment.

Building on contemporary research on therapeutic alliances, alliances might experience ruptures and reparation of these might serve as useful interventions in therapy [28, 29]. In the context of involuntary treatment, the patient might perceive the starting point of involuntary admission as a rupture, thus complicating the working alliances from the start [30]. However, the therapists in this study experienced negotiating working alliances as somewhat easy. Although a number of difficulties might arise underway, findings imply it as possible to achieve and repair working alliances, i.e. solving treatment discussions. Yet, we question, is this because the therapists serve as the main decision maker and, to a certain degree, excludes patients from decision-making processes? Further, we question, whether the therapists are relying on relational aspects, i.e. establishing trust and safety, when agreement seems difficult to achieve? As our results indicate, the mere absence of conflict, when the patient resists from opposing coercive treatment, may be interpreted as equivalent to a good working alliance. If so, the therapists arguably emphasise subjectively chosen aspects of the working alliance rather than considering all components of the concept.

Across the themes observed, patient autonomy opposes therapists’ decisions on expedient treatment. Mol [38] referred to these contradictory absolutes as (1) the *logic of choice* where patients have the freedom to choose between treatment alternatives and (2) the *logic of care* where health professionals assess the need for care and further define and decide on treatment options. She asks: “If it is compared with ‘force’, then ‘choice’ is more often than not a great good. But what about comparing it with ‘care’?” [38, p. xii]. Consistently, the participants expressed a will for patient involvement, yet concluded in what we consider to be in accordance with the *logic of care*. Within this framework, the therapists considered it irresponsible and uncaring not to subject their patients to involuntary treatment due to potential consequences such as worsened mental illness conditions or potential death. This dilemma was concluded by evaluating whether a patient could consent to treatment or not. This finding could be viewed as part of a global trend towards an increasing use of coercive psychiatric interventions both in inpatient and outpatient settings [39]. Further, it could be argued that the increased use of coercion, “prioritises risk management over individual health and social needs [which] is likely to be counterproductive” [39]. Conversely, if the therapists had used the *logic of choice* in its absolute form, involuntary treatment might arguably not be an option. This illustrates extremes of two *logics* discovered in our analysis: involuntary treatment can be life-saving and oppressive whereas voluntary treatment can be self-determinative but with potential consequences of death. Our analysis shows that therapists oscillate between these absolutes or attempt to apply both at the same time by allowing patients to live their lives as long as they are not a danger to themselves or society. It is well established that mental health services, in the context of this study, can be viewed as trying to reconcile interests for patient autonomy and safety for society [40]. However, these interests may not be synergistic; further, they seem to be a great challenge to achieve. For the therapists participating in this study, patient autonomy for those with serious mental illnesses appeared difficult to achieve.

Recovery-oriented practice, in a sense, opposes Mol’s *logic of care*, as a treatment focus is based on first-person definitions of how to live a meaningful life within the context of mental illness [21]. This does not mean that recovery-oriented practice opposes all uses of involuntary treatment, for example, when a person is considered to be suicidal; however, here, emphasis is placed on doing so with respect, dignity and transparency [41]. A central aspect of such processes is in a recovery-oriented practice based on shared decision-making [42–44], and an important implication of our study is the need to develop and implement tools for working with shared decision-making in context of involuntary treatment. As this is clearly a complex issue with people’s opinions and values continuously being shaped by numerous factors such as mental health professionals, their close relations and society at large [45], a high degree of reflexivity [46] is called for in the application of such tools.

Our findings imply that there is an abstract boundary between when and under what circumstances patients’ wishes are considered. It is unknown where this boundary ends, as the decisive conclusion lies with the therapist. This is problematic, as patients who are offered options are more likely to engage in treatment, to join interventions, and to experience better treatment outcomes [47] and studies underscore self-agency as central to recovery processes [48]. It is also problematic that patient inclusiveness is not clearly defined, as this gives an unknown amount of power to the therapist. Recently, the development of the Power Threat Meaning Framework [49] has highlighted how power operates and impacts the lives of those with mental illnesses both within and outside of mental health services. Participants of our study preferred non-coercive interventions, which is in line with findings of a comprehensive study of Norwegian health professionals’ attitudes towards coercive care [50]. At the same time, therapists described limiting treatment options to what they believed to be best for each patient. Taking this final point into account, we find it difficult to conclude that therapists participating in this study are supporting patients as equal agents in shaping treatment plans.

### Limitations

Our findings are shaped by the contexts of the participants interviewed and by the setting in which the study was conducted. The experiences analysed are conclusively based on a health professional’s perspective and do not cover those of the patient participants. This is a limitation of our study, as we examined phenomena depending on a two-part collaboration. Moreover, studies show that therapist assessments of working alliances are less reliable than patient assessments [51]. This might limit the generalisability of our findings to other populations, and further studies must also consider patients’ perspectives. As another possible limitation, our participants might have attributed different meanings to the term “working alliance” (i.e. terms such as “alliance” and “relation” were used synonymously), though Bordin’s definition was explicitly described in the interviews. Data based on different definitions were included, as participants found this meaningful in relation to the examined phenomena. As another limitation, we did not discriminate between different forms of involuntary treatment. There is clearly a difference between different coercive measures such as the use of medication or seclusion, and future research must consider exploring these differences in detail. Furthermore, the participants’ experiences had developed in both inpatient and outpatient contexts in relation to different phases of serious mental illness and to different treatments, thus representing a diverse context. Whilst this allowed us to consider a wide range of therapists’ experiences and views, it also limits our ability to generalise our findings. Finally, the current study uses a qualitative approach to the research question, which brings certain limitations, i.e. a comprehensive data set and assessment of data. Future studies should aspire to include quantitative methods, i.e. developing an operationalisation and scoring the working alliance in involuntary treatment settings.

## Conclusions

We conclude that the therapists exhibited a will to consider their patients’ goals and methods, but only when they were in agreement, and they ultimately made treatment decisions themselves. Further, patient autonomy seems to come second in therapist assessments of needs for care; consequently, we question to what degree the working alliance as a defined concept of mutual agreement is present in the involuntary treatment we investigated.

## Data Availability

The datasets used and analysed for this study are available from the corresponding author on reasonable request.

## Appendix

**Table:**

Interview guide Introduction Can you describe what it is like providing involuntary treatment to people with serious mental illnesses? *Possible follow*-*up questions* What is it *like* executing coercive treatment? Do you experience any differences from other ways of working with patients in therapy? Establishing working alliance in coercive treatment How do you experience establishing contact with people subjected to coercive treatment? *Possible follow*-*up questions on sub*-*themes:* *Therapeutic bond* How do you define «therapeutic bond» ? What is this bond like when the person is subjected to coercive treatment? What do you do to achieve contact with people who are treated involuntary? What do you do to establish a therapeutic bond between you and the patient? Please, feel free to highlight an example. What did you do together? How did you work? Please, feel free to highlight an example where you experienced it as challenging to establish a therapeutic bond What challenges have you met in establishing a therapeutic bond? Have you experienced alliance ruptures? If yes: How did you proceed to solve this rupture? What do you do to make the person feel safe?	*Goals* How do you proceed to find goals with the person subjected to coercive treatment? How do you go about creating agreement between you and the person? How do you include the patient’s goals? Have you experienced any challenges in relation to agreeing on mutual goals? If yes, can you describe these? How do you proceed to solve these? If the person in treatment disagrees with your professional assessment, what do you do? Do you have a specific example? How did you solve this? Has it ever happened that you did not agree with the person subjected to coercive treatment? What do you do in those circumstances? *Methods* What do you do to determine with the person how you might achieve particular goals? What challenges do you face when establishing mutual agreements? If the person disagrees with your professional assessment, what do you do? Do you have a specific example? Elaborate. How did you solve this? Has it ever happened that you did not agree with the person subjected to coercive treatment? If yes, please elaborate. What do you do in those circumstances? Conclusion How could mental health services improve establishing contact with people with serious mental disorders?
